# A Machine Learning Approach to Monitor the Emergence of Late Intrauterine Growth Restriction

**DOI:** 10.3389/frai.2021.622616

**Published:** 2021-03-08

**Authors:** Nicolò Pini, Maristella Lucchini, Giuseppina Esposito, Salvatore Tagliaferri, Marta Campanile, Giovanni Magenes, Maria G. Signorini

**Affiliations:** ^1^ Dipartimento di Elettronica, Informazione e Bioingegneria (DEIB), Politecnico di Milano, Milan, Italy; ^2^ Department of Psychiatry, Columbia University Irving Medical Center, New York, NY, United States; ^3^ Department of Obstetrical Gynaecological and Urological Science, Federico II University, Napoli, Italy; ^4^ Department of Electrical, Computer and Biomedical Engineering, University of Pavia, Pavia, Italy

**Keywords:** late intrauterine growth restriction, machine learning, perinatal medicine, predictive monitoring, support vector machines

## Abstract

Late intrauterine growth restriction (IUGR) is a fetal pathological condition characterized by chronic hypoxia secondary to placental insufficiency, resulting in an abnormal rate of fetal growth. This pathology has been associated with increased fetal and neonatal morbidity and mortality. In standard clinical practice, late IUGR diagnosis can only be suspected in the third trimester and ultimately confirmed at birth. This study presents a radial basis function support vector machine (RBF-SVM) classification based on quantitative features extracted from fetal heart rate (FHR) signals acquired using routine cardiotocography (CTG) in a population of 160 healthy and 102 late IUGR fetuses. First, the individual performance of each time, frequency, and nonlinear feature was tested. To improve the unsatisfactory results of univariate analysis we firstly adopted a Recursive Feature Elimination approach to select the best subset of FHR-based parameters contributing to the discrimination of healthy vs. late IUGR fetuses. A fine tuning of the RBF-SVM model parameters resulted in a satisfactory classification performance in the training set (accuracy 0.93, sensitivity 0.93, specificity 0.84). Comparable results were obtained when applying the model on a totally independent testing set. This investigation supports the use of a multivariate approach for the *in utero* identification of late IUGR condition based on quantitative FHR features encompassing different domains. The proposed model allows describing the relationships among features beyond the traditional linear approaches, thus improving the classification performance. This framework has the potential to be proposed as a screening tool for the identification of late IUGR fetuses.

## Introduction

Antenatal fetal heart rate (FHR) is a widely used tool to monitor fetal wellbeing (Chen et al., 2011). The assessment of fetal heart rate variability (HRV) has been reported to inform on the functional state of the autonomic nervous system (ANS), thus providing an indication on the fetal development throughout pregnancy. In the context of fetal pathological states, intrauterine growth restriction (IUGR) is one of the most relevant complications of pregnancy and it has been reported to alter HRV (Huhn et al., 2011; Signorini et al., 2020b). IUGR is associated with a decreased rate of fetal growth, which is the result of an abnormal supply of maternal nutrients and placental transfer to the fetus. IUGR is a pathological fetal state characterized by an increased mortality and/or morbidity (Rosenberg, 2008; Smith et al., 2013; Sharma et al., 2016). The two phenotypes of IUGR (early and late) can be identified based on onset, evolution, Doppler parameters modifications, and postnatal outcome (Esposito et al., 2019).

In this paper we will focus on a population of late IUGR which is a condition with substantial increased prevalence if compared to early IUGR (Villar et al., 2014; Gordijn et al., 2016). The main cause for the insurgence of late IUGR is fetal hypoxemia/hypoxia secondary to placental insufficiency. Moreover, it is often associated with multiple placental anomalies that by contrast have less influence on placental resistance. Therefore, the umbilical Doppler indices are often unaffected, thus making the diagnosis of late IUGR more difficult, due to the large variability of fetal parameters on growth charts in the third trimester (Mureşan et al., 2016). Late IUGR is suspected when the fetal growth curve slows down or does not physiologically increase as a function of gestational age. Undetected IUGR in the third trimester of pregnancy represents the main cause of unexplained stillbirths in low-risk pregnancies, thus better antenatal diagnosis and treatment and timely delivery could diminish the risks significantly (Warland and Mitchell, 2014). In order to investigate identification of late IUGR through FHR analysis, we used the cardiotocography (CTG), which combines the measure of FHR through a Doppler ultrasound probe with the detection of uterine contractions using a pressure sensor. Although CTG analysis is still performed visually in the majority of Ob-Gyn clinical settings [following guidelines edited by national and international scientific societies, such as the International Federation of Gynecology and Obstetrics (FIGO) (FIGO, 1986)], a progressive transition to computerized approaches has been reported in recent years. Computerized systems are able to extract FHR parameters from multiple domains [time domain and frequency domain, complexity/nonlinear methodologies (Task Force of The European Society of Cardiology and The North American Society of Pacing and Electrophysiology, 1996)] and represent the initial step toward multiparametric and multidimensional FHR analyses able to benefit from machine learning algorithms. As a matter of fact, the FHR regulation is the result of multiple and diverse neurological feedback loops, hormones, and various external factors, thus resulting into complex temporal dynamics, which are usually missed by the simple visual inspection. Additionally, previous studies have shown the strength of a multivariate framework in detecting fetal acidemia (Spilka et al., 2017) and a previous paper from our group used machine learning approaches to diagnose IUGR, but mainly focusing on the early phenotype (Signorini et al., 2020b).

In this study, classical FHR features were complemented with advanced nonlinear features and subsequently employed to train several machine learning algorithms for the detection of late IUGR in a database of 262 fetuses. Results highlighted the enhanced performance of nonlinear features over traditional parameters and the significant improvement in specificity and sensitivity of multiparametric machine learning approaches over univariate analysis. Furthermore, by utilizing an interpretable variant of support vector machines, we were able to identify the features that contributed the most to classification accuracy. This implementation provided meaningful and interpretable results with the potential of their translation into clinical practice.

## Material: Database and Preprocessing

### Dataset

Antepartum FHR recordings were collected at the Azienda Ospedaliera Universitaria—Federico II (Napoli, Italy). Data analyzed in the investigation were collected as part of the routine clinical examinations administered to pregnant women by the Italian healthcare system. Pregnant women signed informed consent for the utilization of their data for research purposes. The ethical committee and the IRB of Azienda Ospedaliera Universitaria—Federico II, Napoli, Italy, approved the enrollment of pregnant women as participants in the study and the utilization of the routine examination for research purposes. Traces were recorded in a controlled clinical environment, with participants lying supine on a bed undergoing a standard nonstress test protocol. Data were acquired using Philips cardiotocography (CTG) fetal monitor Avalon FM30 connected to a computer. The device employs an autocorrelation technique to compare the demodulated Doppler signal of a given heartbeat and the subsequent one. The resulting resolution for beat detection is below 2 ms. The derived CTG signal consists of a series of FHR values sampled at 2 Hz and expressed in beats per minute (bpm). Additionally, each FHR sample is accompanied by an indication of signal quality: optimal, acceptable, or insufficient based on the results of autocorrelation technique. We excluded pregnant women with maternal health conditions known to affect FHR regulation (gestational diabetes, preeclampsia, and hypertension), psychiatric medication use during pregnancy (SSRIs, antidepressants, classic antipsychotics, atypical antipsychotics, mood stabilizers, stimulants, antianxiety medications, or anticonvulsants), any recreational drug use during pregnancy, and congenital heart anomalies. Fetuses with congenital heart anomalies and genetic disorders were also excluded. The cohort analyzed in this work comprised 102 late intrauterine growth restriction (IUGR) fetuses and 160 healthy fetuses matched for GA at the first CTG examination. Fetuses in both groups underwent a routine ultrasound examination at approximately 34 weeks GA which did not exhibit any alteration in fetal growth or abnormalities in neither the middle cerebral artery nor the ductus venosus Doppler velocimetry. Once subsequently admitted for a CTG recording at 37.54 ± 0.77 (mean ± std) weeks, fetuses in the healthy group did not show any abnormality in the FHR trace, whereas IUGR fetuses (admitted at 36.94 ± 0.59 weeks) did present irregularities in their CTG recordings. A concurrent ultrasound examination showed alteration in both growth and Doppler profiles in this group of fetuses. The clinical definition of late IUGR adopted in this work reflects the guidelines reported in Gordijn et al. (2016). The adopted classification combines standalone information from multiple domains—gestational age, congenital abnormalities, absolute size measurements, and functional parameters—as well as their interactions (Gordijn et al., 2016). Each prenatal fetal condition was verified after delivery to confirm group membership previously suspected at the CTG timepoint. The length of the CTG recordings considered in this study is 40 min in both populations. This guaranteed that FHR data were acquired during both activity and quiet periods for any given fetus included in the study. Participants were not included in the analysis if their associated recordings were of insufficient duration and/or with <30 usable 1-min epochs and/or <10 3-min epochs (see next section for definition of epochs). Clinical data of the analyzed populations are reported in Table 1. Fetuses in the healthy group were characterized by higher birthweight, Apgar score 1 min, and rate of spontaneous vaginal delivery compared to the considered IUGR population. Similar results have been reported in other populations of early (Stampalija et al., 2015) and late (Esposito et al., 2019) IUGR fetuses.

**TABLE 1:** Clinical data for the healthy and IUGR populations.

	Healthy *n* = 160	IUGR *n* = 102
GA at CTG (weeks)^†^	37.54 ± 0.77	36.94 ± 0.59
Maternal age (years)	32.23 ± 5.16	32.36 ± 5.82
Birthweight (g)^†^	3,311.62 ± 373.87	2,038.40 ± 348.15
Umbilical cord pH^†^	7.28 ± 0.08	7.32 ± 0.06
Fetal sex (male)	55.00%	46.08%
Apgar score 1 min >7^†^	91.88%	78.43%
Apgar score 5 min >7	100.00%	98.04%
Mode of delivery^†^	59.38% vaginal	28.43% vaginal
	40.62% caesarean	71.57% caesarean

Expressed as mean ± standard deviation or number (%). ^†^denotes a significant difference between healthy and IUGR fetuses, *p* < 0.05.

### Fetal Heart Rate Time Series and Preprocessing

The equipment used to record the data under investigation provides each FHR sample with an indication of signal quality: optimal (green), acceptable (yellow), or insufficient/absent (red) based on the results of the embedded autocorrelation procedure utilized to extract the signal itself (Lawson et al., 1983; Signorini et al., 2003). The first preprocessing step toward the computerized analysis of the acquired traces consisted in subdividing each FHR recording in shorter segments of length 120 points (60 s) or 360 points (180 s). The choice of 1-min or 3-min subintervals is related to the different time scales on which CTG-derived features are computed, whose procedure will be described in the following sections. Subsequently, segments including more than five consecutive red-quality points or more than 5% of red-quality samples (6 FHR values out of 120 points per subinterval or 18 FHR values out of 360 points per subinterval) were discarded in further analysis. Lastly, isolated insufficient-quality points were substituted, through a moving average procedure, with the average of the nearest five FHR points. For an in-depth description of the preprocessing steps adopted in this investigation, see previous publications by our group (Arduini et al., 1993; Signorini et al., 2003; Magenes et al., 2007).

## Methods: Features and Radial Basis Function Support Vector Machines

### Features

**FIGURE 1:**
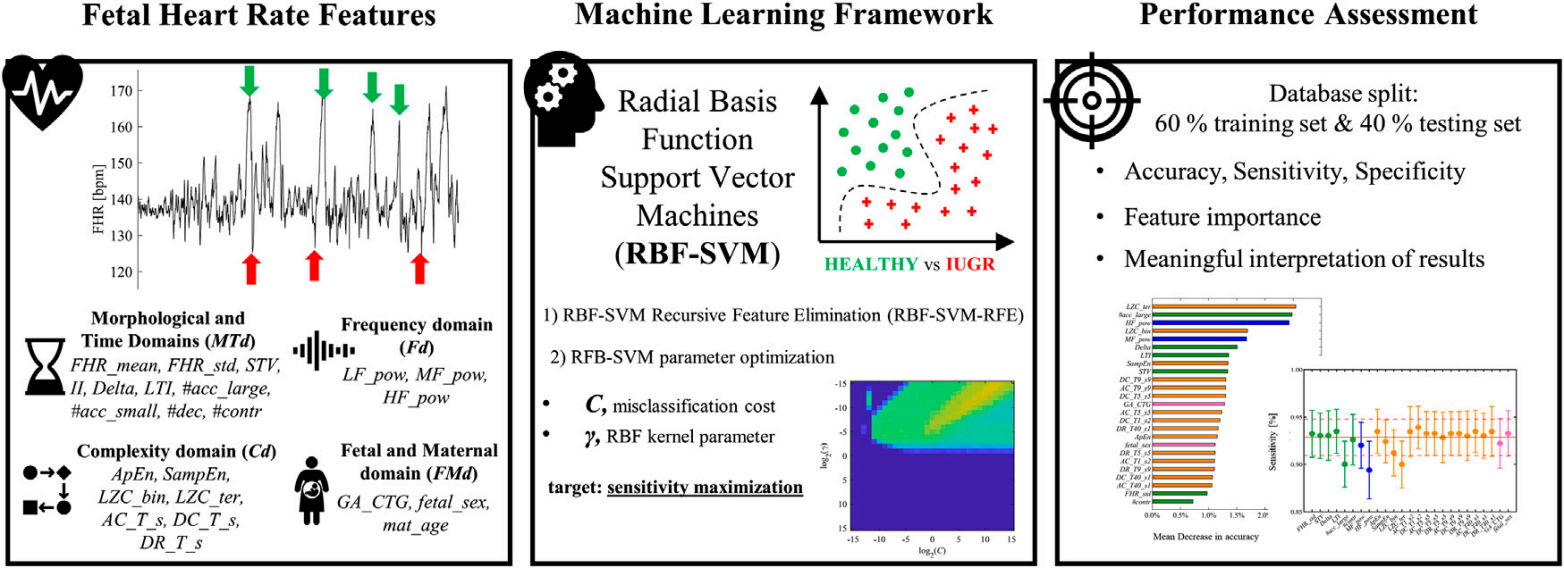
A schematic depiction of the machine learning framework to monitor the emergence of late intrauterine growth restriction. The starting point was the extraction of fetal heart rate physiology features. Morphological and Time Domains *(MTd)* – mean, standard deviation of entire FHR signal *(FHR_mean, FHR_std)*; Short Term Variability *(STV)*; Interval Index *(II)*; Delta; Long Term Irregularity *(LTI)*; large accelerations (more than 15 beats per minute over the baseline lasting 15 s or more, indicated by green arrows) *(#acc_large)*; small accelerations (fewer than 15 beats per minute) *(#acc_small)*; decelerations (*#dec,* indicated by red arrows); contractions *(#contr)*. Frequency domain *(Fd)* – power in the low frequency (LF) band *(LF_pow)*; power in the movement frequency (MF) band *(MF_pow)*; power in the high frequency (HF) band *(HF_pow)*. Complexity domain *(Cd)*—Approximate Entropy *(ApEn)*; Sample Entropy *(SampEn)*; binary, ternary Lempel Ziv Complexity *(LZC_bin, LZC_ter)*; Average Acceleration, Deceleration Capacities *(AC_T_s, DC_T_s)*; Deceleration Reserve *(DR_T_s)*. Fetal and Maternal domain *(FMd)* – GA at the recording *(GA_CTG)*; fetal sex *(fetal_sex)*; maternal age *(mat_age)*. Radial basis function support vector machine (RBF-SVM) model was developed to discriminate between healthy and late IUGR fetuses. The ensemble of previously described features was reduced by means of Recursive Feature Elimination technique (RBF-SVM-RFE). The optimization of RBF-SVM parameters *C* and *γ* aimed at maximizing sensitivity is shown in the bottom graph of the Machine Learning Framework panel. Performance was assessed by splitting the database of 160 healthy and 102 late IUGR fetuses in a training (60%) and independent testing set (40%). Several figures of merit were computed. Additionally, the proposed implementation of RBF-SVM allowed deriving interpretable feature importance ranking and standalone feature contribution to accuracy, sensitivity, and specificity (left and right (only sensitivity shown) graphs in the Performance Assessment panel).

The present contribution focuses on building a machine learning-based screener of late IUGR pathology fed with a set of FHR features rather than a single feature design-oriented approach. Thus, the employed features were selected on the basis of the a priori knowledge on various quantifiers of fetal ANS dynamics in different domains, complemented by fetal and maternal information. Figure 1 reports a schematic workflow for the framework implemented in this work.

#### Morphological and Time Domains

Morphological and time domain features represent the computerized and automated extraction of FIGO guidelines from FHR recordings, in terms of baseline evolution, accelerations/decelerations, and variability. Starting from the identification of FHR baseline [by means of Mantel’s approach (Mantel et al., 1990)], it is possible to derive the automatic counts of large accelerations (more than 15 beats per minute over the baseline lasting 15 s or more) (*#acc_large*), small accelerations (fewer than 15 beats per minute) (*#acc_small*), decelerations (*#dec*), and contractions (*#contr*) (Rabinowitz et al., 1983; FIGO, 1986). FHR variability features are derived from FHR signal excluding events of accelerations and decelerations. Specifically, the overall variability is quantified by the mean and standard deviation of entire FHR signal (*FHR_mean* and *FHR_std*). Short Term Variability (*STV*), Interval Index (*II*), and *Delta* provide estimates of short term FHR variability considering 1-min FHR intervals. Long Term Irregularity (*LTI*) quantifies variability on a longer time scale (3-min FHR intervals). A more comprehensive description and characterization of the employed FHR variability features can be found in previous publications by our group (Arduini et al., 1993; Signorini et al., 2003; Magenes et al., 2007).

#### Frequency Domain

Power Spectral Density (PSD) is a largely exploited method for HRV frequency analysis. It decomposes the signal power in oscillatory components which are an indirect measure of ANS modulation over the cardiac system. PSD is computed employing autoregressive (AR) modeling, specifically by Levinson-Durbin algorithm. Based on previous findings, three specific frequency bands of interest can be identified, namely, low frequency (LF) (0.03–0.15) Hz; movement frequency MF (0.15–0.5) Hz; high frequency HF (0.5–1 Hz), which quantifies the different branches of ANS modulation (Signorini et al., 2003; Faes et al., 2015; Spilka et al., 2017). The FHR signal undergoes an automatic decomposition into a sum of sinusoidal contributions identified by their central frequencies and the associated amount of power, thus obtaining the power in the LF band (*LF_pow*), MF band (*MF_pow*), and HF band (*HF_pow*) for each 3-min FHR segment.

#### Complexity Domain

The application of nonlinear methodologies to investigate FHR variability has demonstrated its usefulness in predicting fetal wellbeing in several investigations (Signorini et al., 2003; Spilka et al., 2012; Gonçalves et al., 2018). In the context of early IUGR detection, Lempel Ziv Complexity (LZC) (Lempel and Ziv, 1976) has been previously reported to have enhanced discriminative power in both univariate (Ferrario et al., 2007) and multivariate (Signorini et al., 2020b) approaches, considering binary (*LZC_bin*) and ternary (*LZC_ter*) alphabets. Additional measures of complexity such as Approximate Entropy (*ApEn*) (Pincus and Viscarello, 1992) and Sample Entropy (*SampEn*) (Lake et al., 2002) have been employed for the quantification of ANS profiles in the perinatal period. The above described features are computed for each 3-min FHR segment. The last nonlinear technique is Phase Rectified Signal Averaging (PRSA) (Bauer et al., 2006). Average Acceleration and Deceleration Capacities (*AC* and *DC*) are among the various parameters which can be derived from the PRSA curve (Fanelli et al., 2013). More recently, Deceleration Reserve (*DR*) (Rivolta et al., 2019) was defined as the simple summation of *AC* and *DC* and it has been shown to achieve enhanced performance in detecting fetal hypoxia compared to *AC* and *DC* standalone parameters in the context of intrapartum FHR recordings. Regarding the specific implementation of these methodologies in this work, for the computation of *LZC_bin* and *LZC_ter* the factor value (*p*) was set to zero, whereas for entropy computation the length of the pattern (*m*) and tolerance (*r*) were set equal 1 and 0.1, respectively, accordingly with the prior knowledge on their application for fetal investigations (Faes et al., 2015; Gonçalves et al., 2018; Signorini et al., 2020b). On the other hand, a technical aspect that complicates the physiological understanding of PRSA-derived features is their dependence on three parameters, namely, *T*, *s*, and *L*. Different combinations of the former parameters allow gaining insight about the ANS branches separately. In this work, *AC*, *DC*, and *DR* were computed considering *T* = 1 and *s* = 2, *T* = 5 and *s* = 5, *T* = 9 and *s* = 9, *T* = 40 and *s* = 1, and *L* was constant and equal 100.

#### Fetal and Maternal Domain

The evolution of fetal ANS regulation throughout pregnancy has been extensively investigated in regard to GA, sex, and various aspects (Giuliano et al., 2017; Gonçalves et al., 2017, 2018). This evidence is consistently reported among *MTd*, *Fd*, and *Cd* features. To address this issue, GA at the recording (*GA_CTG*), fetal sex (*fetal_sex*), and maternal age (*mat_age*) are included in the machine learning analyses.

#### Feature Preprocessing

The time series of each parameter was averaged throughout the recording to derive a single set of features for each subject. At this step, features were preprocessed for outliers [Winsorization in the interval (*Q*_1_ – 3*IQR*, *Q*_3_ + 3*IQR*), where *Q*_i_ is defined as the *i*th quartile and IQR = *Q*_3_ – *Q*_1_]. Lastly, features were standardized across the entire population to obtain zero mean and unitary variance distributions.

### Radial Basis Function Support-Vector Machines

#### From Linear to Radial Basis Function Support Vector Machines

Support Vector Machines (SVM) are a class of machine learning algorithms highly exploited for the purposes of data classification and regression. As a general consideration, SVM aim to derive a model learned on the training data, which is able to predict the target values contained in the test data (Cortes and Vapnik, 1995; Guyon et al., 2002; Hsu, Chang and Lin, 2003). Given a training set of labeled instance pairs (***x***_i_, *y*_i_), *i* = 1,…,*l*, where ***x***_i_ ∈ ℝ^*n*^ and *y* ∈ {1,−1}^*l*^, *l* is equal to the number of observed pairs, *n* is the dimensionality of the feature space, and *y* corresponds to healthy/late IUGR binary classification assigned to each participant. SVM searches for the optimal hyperplane ***w*** ^*T*^ *ϕ* (***x***_*i*_) *+ b*, which maximizes the separation margin between the two classes by solving an optimization problem. In the context of classical SVM, such function is linear; thus the corresponding term reads ***w*** ^*T*^ ***x***_*i*_ *+ b*, which translates into a linear separating hyperplane. *C* > 0 is the so-called penalty parameter of the error term. *C* controls the tradeoff between misclassification and data sparsity. Large values of *C* constrain the optimization procedure to derived smaller-margin hyperplane if such boundary contributes to the training points classified correctly. Conversely, a smaller value of *C* causes the optimizer to search for larger margins, even if the derived hyperplane misclassifies more observations. Classical SVM promote data sparsity given only few subjects contribute to the margin determination at the expenses of involving all the features, thus being affected by the curse of dimensionality (Hsu et al., 2003). To address the described issues, we propose to employ a more efficient kernel function: Radial Basis Function SVM (RBF-SVM) (Hsu et al., 2003), and a novel feature elimination algorithm, namely, RBF-SVM Recursive Feature Elimination (RBF-SVM-RFE) (Liu et al., 2011). The main shortcoming of classical SVM is the constraint of describing the relationship between the class labels and the features as linear. On the opposite, the kernel of RFB-SVM maps observations into a higher dimensional space, thus allowing for a nonlinear relationship between observations and attributes. In this scenario, the function *ϕ* can be expressed according to (Eq. 1):

$$K\left(x_{i},x_{j}\right)=e^{-\gamma x_{i}-x_{j}^{2}},\;\;\;\gamma >0_{\;},$$ (1)

where *K* is called kernel function, and the parameter *γ* defines the radius of influence of a given training example. Specifically, low values of *γ* code for far influence and a very broad decision region, whereas high values of *γ* result in the opposite. Furthermore, it can be shown that RFB kernel is equivalent to the linear one for some combinations of (*C*, *γ*) (Lin and Lin, 2003). RBF-SVM are suitable to be employed in the presented study given the well-documented nonlinear relationship between several features and the target binary outcomes: healthy or IUGR fetuses (Signorini et al., 2003; Spilka et al., 2017; Gonçalves et al., 2018).

#### Radial Basis Function Support Vector Machine Recursive Feature Elimination

Linear SVM Recursive Feature Elimination (SVM-RFE) is a largely exploited category among the wrapper models (Kohavi and John, 1997) which performs feature selection (Guyon et al., 2002). Wrapper methodologies are computationally demanding but they exhibit enhanced performance compared to filter approaches (Sun, 2007). If SVM-RFE allows deriving an interpretable feature ranking, the same is not valid when considering nonlinear SVM (as for RBF-SVM). This relates to the fact the mapping function *ϕ* is unknown; thus the vector ***w*** cannot be explicitly computed and consequently cannot be used to rank features as for SVM-RFE. In this work, we employed a recent extension of SVM-RFE which performs feature elimination in the context of nonlinear SVM, namely, RBF-SVM Recursive Feature Elimination (RBF-SVM-RFE) (Liu et al., 2011). In a nutshell, RBF-SVM-RFE expands RBF kernel into its Maclaurin series. The weight vector ***w*** is derived from the series by computing the contribution made to the classification hyperplane of each feature. The algorithm allows ranking features by their relative importance starting by including all features and progressively eliminating each of them until all attributes are ranked. Moreover, RBF-SVM-RFE allows deriving the most informative subset of feature among all possible permutations of the original set. A comprehensive and rigorous description of the algorithm can be found in Liu et al. (2011).

### Performance Assessment

Performance is quantified in terms of the area under receiver-operation-characteristic (ROC) curve (AUC), sensitivity (SE), and specificity (SP). In the context of supervised machine learning approaches as for RBF-SVM, it is usually required to perform the following: 1) make use of cross-validation (CV) to identify the best pair of parameters *C* and *γ*; 2) train the whole training set using the previously identified *C* ^opt^ and *γ* ^opt^ and evaluate the performance; 3) test the validity, replicability, and stability of the model on a new set of observations which have never been used in the training phase. The prediction accuracy obtained from the unknown observations is thought to reflect in a more precise way the classification performance of the trained algorithm. In the context of this work, the training set was obtained by including 60% of the original dataset and utilized to perform task 1) and task 2), whereas the remaining 40% was used to derive the independent test set and employed in task 3). The operation of testing the model performance uniquely on validation dataset does not guarantee unbiased results as the model is fitted on the training dataset while tuning model hyperparameters. On the contrary, the utilization of an independent test has been shown to provide an unbiased evaluation of the final model. The ratio between healthy and IUGR (∼1.5:1) was maintained constant in both sets. *C* ^opt^ and *γ* ^opt^ were derived by performing a grid search on *C* and *γ* using cross-validation. Specifically, several pairs of (*C*, *γ*) were tested and the one achieving the best cross-validation figure of merit was chosen. Exponentially growing sequences of *C* and *γ* were employed in a the grid search framework (Hsu et al., 2003; Spilka et al., 2017) along with a 10-Fold CV repeated 5 times on the training set to identify *C* ^opt^ and *γ* ^opt^. In this work, SE was identified as the figure of merit to be maximized to the aim of deriving a screening tool suitable to be employed in clinical practice for the identification of late IUGR. Despite its straightforward implementation compared to more advanced methodologies, a grid search approach has the advance of avoiding approximations by performing an exhaustive parameter search. Additionally, it can be easily parallelized since each (*C*, *γ*) pair is independent. On the opposite, iterative processes can hardly be run simultaneously (Hsu et al., 2003).

## Results

### Univariate Analysis

For benchmark, the performance in discriminating healthy vs. IUGR fetuses for each of the previously described features was computed. Specifically, a set of logistic regression models were trained including each attribute individually. The optimal cut-off (*c*) for a given feature was derived by the maximization of Youden Index (Fluss et al., 2005) defined as *J* = *max*_*c*_ (*SE* (*c*) + *SP* (*c*) – 1). *J* allows computing the optimal *c* and consequently the corresponding SE (*c*), SP (*c*), and AUC (*c*) values. Table 2 reports the ten best performing features ranked by their AUCs. *Cd* features yield the best univariate classification results, followed by *MTd* ones. Notably, neither *Fd* nor *FMd* attributes have a significant individual power. The selected features clearly point to the importance of more sophisticated analyses of FHR, rather than the traditional time and frequency approaches. Despite satisfactory values of AUC, the corresponding SE and SP suggest the need for a multivariate framework in order to improve and balance the overall performance. Prior to multivariate classification, correlation among all pairs of features was performed: 1) short and longer term *MTd* features were moderately correlated; 2) short term variability measured in the different domains: *ApEn*, *SampEn*, *HF_pow*, *LZC_bin*, and *LZC_ter* was highly correlated as expected given their definitions; 3) *ApEn*, *SampEn*, *LZC* parameters did not exhibit any relationship with PRSA-derived features; 4) *AC*s and *DC*s at different scales exhibited marked negative correlations; 5) *DR*s were weakly positive correlated with the corresponding *DC*s but not with *AC*s.

**TABLE 2:** Univariate performance.

	AUC	Sensitivity	Specificity
LZC_bin (bits)	0.78	0.78	0.68
LZC_ter (bits)	0.78	0.88	0.57
#acc_large	0.72	0.84	0.48
AC_T9_s9 (bpm)	0.68	0.76	0.50
AC_T5_s5 (bpm)	0.67	0.66	0.58
FHR_std (bpm)	0.66	0.72	0.55
#Contr	0.65	0.66	0.58
LTI (ms)	0.63	0.66	0.58
Delta (ms)	0.62	0.83	0.40
STV (ms)	0.61	0.98	0.21

Feature cut-offs associated with the optimal values of sensitivity and specificity are derived based on Youden’s index maximization.

### Multivariate Analysis

The performance of several machine learning classifiers was tested against the proposed RBF-SVM methodology. An exhaustive description of the employed techniques is reported in a previous publication by our group (Signorini et al., 2020b). Following the procedure illustrated by Zhang et al. (Zhang et al., 2002), the ROC curves associated with these methodologies were independently compared to the results of RBF-SVM model in a paired design. Results showed that all the tested techniques were statistically inferior to the RBF-SVM model. Nonetheless, features were ranked similarly among the tested machine learning classifiers, supporting the robustness of the proposed physiology based heart rate indices.

#### Feature Selection

The original set of features comprised *n* = 32 attributes, of which *n* = 10 from *MTd*, *n* = 3 from *Fd*, *n* = 16 from *Cd*, and *n* = 3 from *FMd*. The first step prior to multivariate analysis was to reduce the feature space according to RBF-SVM-RFE, as described in the Methods section. The minimum and maximum allowed numbers of features for each subset were *n* = 1 and *n* = 32, respectively. Among the tested subsets, the selected one consisted of 25 retained features and seven eliminated. Specifically, the features with the least squared weights were *FHR_mean*, *II*, *#acc_small*, *#dec*, *LF_pow*, *DR_T1_s2*, and *mat_age*. This result was in accordance with the findings for the univariate analysis. Consistently, the dropped attributes exhibited poor discriminative performance as standalone parameters. Additionally, the results of the correlation analysis for *FHR_mean*, *II*, and *mat_age* highlighted their independence of any other variable included in this analysis. *LF_pow* and *DR_T1_s2* were highly correlated with frequency and PRSA-extracted indices; thus it is likely that their contribution in classification resulted as limited. Lastly, *#acc_small* and *#dec* did not exhibit substantial variations in the two groups.

#### Radial Basis Function Support Vector Machine Parameter Optimization

**FIGURE 2:**
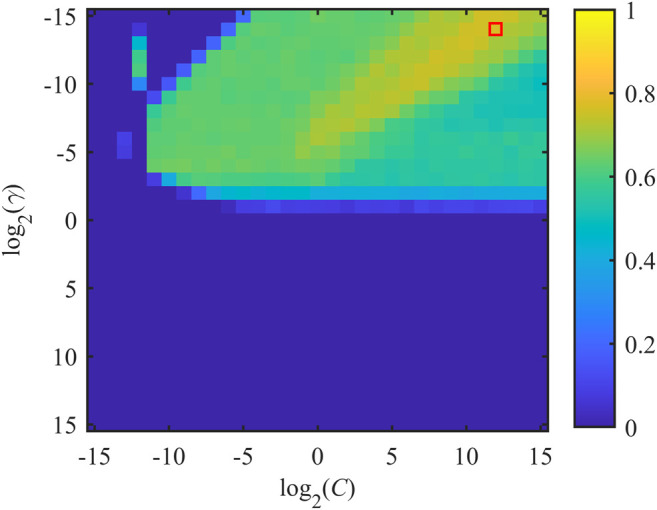
Distribution of *J* as a function of misclassification cost *C* and SVM-RBF kernel parameter *γ*. *x*- and *y*-axes are expressed in logarithmic units for better interpretability of the adopted grid search. *J* ^opt^ is achieved by considering the pair (*C* ^opt^, *γ* ^opt^), which is indicated by the red box.

Various pairs of (*C*, *γ*) were tested to identify the combination (*C* ^opt^, *γ* ^opt^) which corresponded to the maximization of the figure of merit *J*. Each value of *J* was obtained by training the model on the whole set of selected features employing a 10-Fold CV scheme, repeated 10 times. Exponentially growing sequences of *C* = *γ* = 2^−15^, 2^−14^,…, 2^14^, 2^15^ were adopted as practical implementation of the RBF-SVM grid search previously described. The distribution of *J* as a function of *C* and *γ* is shown in Figure 2. About half of the tested pairs (corresponding to positive exponents of *γ*) resulted in an unsatisfactory performance (*J* = 0), which is mapped in the lower half of the plane displayed in Figure 2. The remaining portion of the investigated two-dimensional space is associated with more satisfactory values of SE and SP. Specifically, the optimal combination was achieved by setting *C* ^opt^ = 2^12^ and *γ* ^opt^ = 2^−14^. In this case, *J* ^opt^ was equal to 0.7682 and the corresponding SE and SP were equal to 0.9287 and 0.8395, respectively. Noticeably, SE associated with the reported *J* ^opt^ is the highest achieved for the presented parameter searching. On the other hand, the best SP was equal to 0.8881 but the corresponding SE was 0.7467 (*C* ^opt^ = 2^5^ and *γ* ^opt^ = 2^−15^), thus being unsatisfactory from the perspective of building a screening tool.

#### Performance Assessment on Training and Testing Sets

The pair *C* ^opt^ = 2^12^ and *γ* ^opt^ = 2^−14^ was employed as optimal set of parameters for the final adopted model. This was learned on the training set by a 10-fold CV scheme repeated 10 times, including the restricted set of selected features. The resulting AUC was 0.9277 (0.9109, 0.9445), corresponding to SE equal 0.9287 (0.9095, 0.9479) and SP equal 0.8395 (0.8024, 0.8766). Results are reported as mean and 95% confidence interval. A main drawback of the proposed pipeline is the opportunity for overfitting the model on the training data. The practice of testing the derived model on a validation set aims at evaluating its robustness and insensitivity to overfitting. As previously described, the validation set encompasses 40% of the original dataset with the requirement of a similar ratio of healthy vs. IUGR cases. The model tested on the validation set achieved a close agreement with the one obtained on the training data. In detail, classification accuracy was 0.8462 (0.7622, 0.9094) and the associated values of SE and SP were 0.8438 and 0.8500, respectively. The resulting performance did not exhibit a drastic decrease of AUC, SE, or SP, strengthening the validity of the proposed model as a screening tool. This assumption was highlighted by additional figures of merit such Positive Predicted Value (PP V), 0.9000, and Negative Predictive Value (NPV), 0.7727.

#### Feature Importance

**FIGURE 3:**
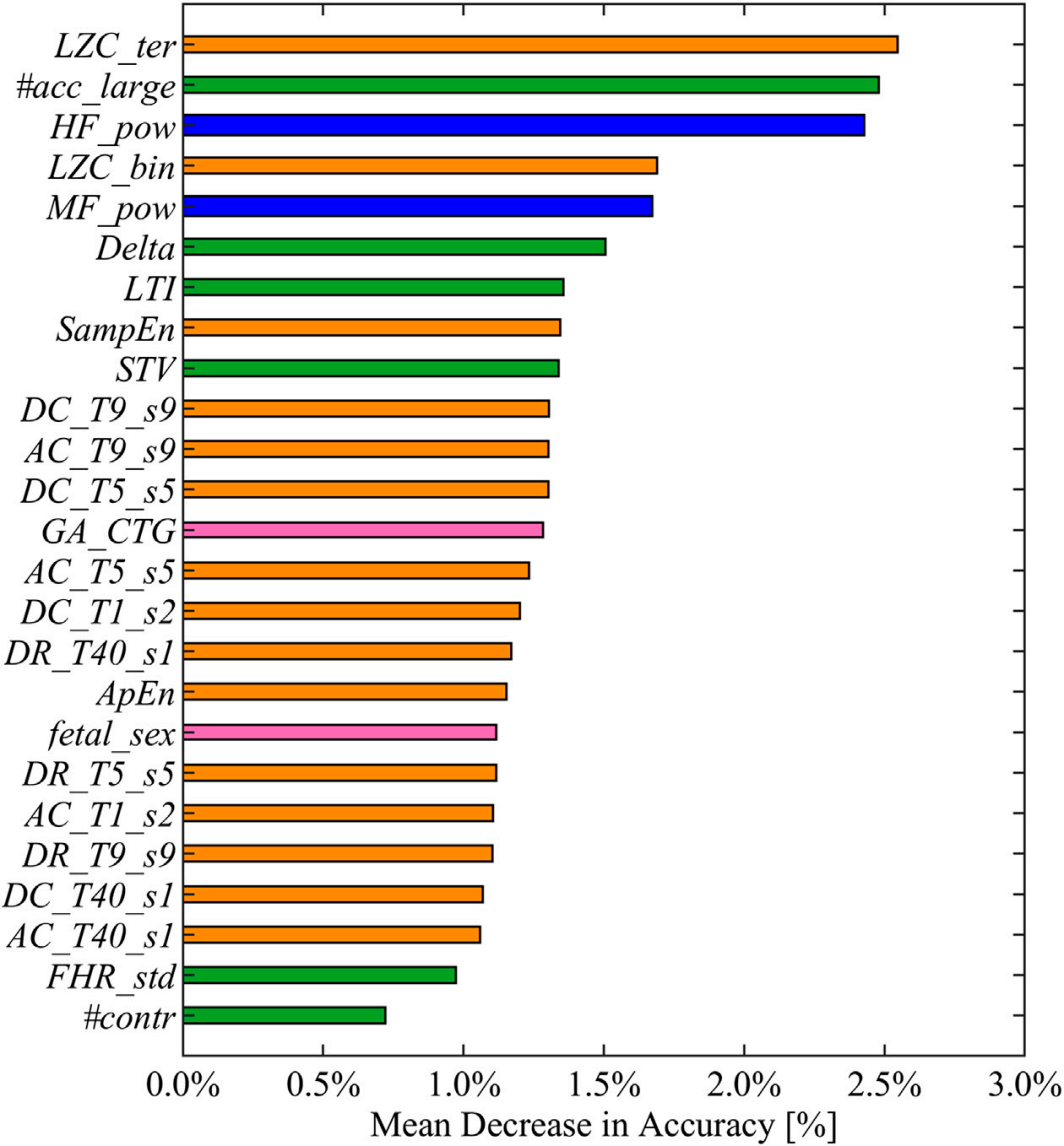
Feature ranked by mean decrease in accuracy in descending order from top to bottom. Mean decreases in accuracy computed as the difference between the optimal accuracy (obtained by including the entire set of selected features in the training set) and the models learned excluding each feature alternatively. The displayed colors code for the different feature domains: green for *MTd*, blue for *Fd*, orange for *Cd*, and pink for *FMd*.

**FIGURE 4:**
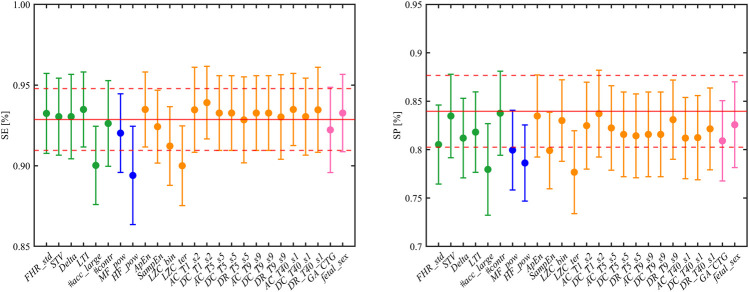
Left and right panels show the resulting SE and SP (mean and CI) when each feature is alternatively excluded from the trained model. The reference values of SE and SP are reported in red, solid and dashed lines correspond to mean and CI, respectively. The displayed colors code for the different feature domains: green for *MTd*, blue for *Fd*, orange for *Cd*, and pink for *FMd*.

The main advantage in employing interpretable fetal heart rate features becomes evident for the purpose of providing meaningful machine learning findings. Specifically, the combination of heart rate attributes and RFB-SVM-RFE allows investigating the relative influence of each attribute toward classification. The results of described approach are displayed in Figures 3, 4. The operation of ranking features according to the weight vector ***w*** was also found to reflect the mean decrease in accuracy of classification when a given feature was removed from the original set employed in the training phase, as shown in Figure 3. The features that, when removed, generated the biggest decrease in accuracy were found to belong to different domain, namely, *LZC_ter* for *Cd*, *#acc_large* for *MTd*, and *HF_pow* for *Fd*. Additionally, the reported mean decrease in discriminative power appeared limited if compared to the reference accuracy achieved in the training set. At the same time, the associated SE and SP highlighted a more pronounced decrease in performance as displayed in Figure 4. The feature specific decreases in SE shown in the left-hand panel in Figure 4 were highly correlated with the results reported in Figure 3. In fact, *LZC_ter*, *#acc_large*, and *HF_pow* accounted for the greatest decrease in SE, whereas SE stayed stable once the remaining features were removed from the model. SP exhibited a similar behavior as reported in the right-hand panel in Figure 4. The described evidence suggests that the employed features contribute similarly to SE and SP. Based on these results, we can conclude that both SE and SP appear as robust figure of merit in the context of the proposed model.

## Discussion

In this investigation, we provided evidence for the successful application of a machine learning framework for the identification of late IUGR condition based on a single routine CTG examination. Starting from the unsatisfactory results of traditional univariate analysis (as reported in Table 2) we proposed an interpretable RBF-SVM model to be employed as screening tool in a clinical setting. The potential of early identification of late IUGR represents a noticeable step toward a better clinical management aimed at improving fetal outcome (Rosenberg, 2008). Discussing the model performance, the achieved values of AUC, and the associated SP and SE demonstrated the consistent ability of the proposed methodology to discriminate healthy vs. late IUGR fetuses in the training and in the validation set. This result is a consequence of the accurate tuning of model parameters (*C*, *γ*) designed to prevent overfitting. The proposed grid search for the optimal pair *C*, *γ* aimed at balancing the tradeoff between the values of model variance and bias. As a general consideration, high values of the misclassification cost (*C*) contribute to hard margin, thus forcing the model to a stricter interpretation of training data, potentially resulting in overfitting the training data. On the opposite, small values of gamma (*γ*) lead to low bias and high variance models. In this work, the selected pair consistently points to a high variance and low bias model. This translates in a separation hyperplane characterized by shaper boundaries and a strong penalization of misclassification error, suitable for the screening tool-oriented applications. Additionally, the absence of a potential bias toward overfitting is supported by the presented results on the validation set.

A crucial aspect for the clinical application of the presented model was the possibility to interpret the data-driven results assessing the features importance. The most noticeable advantage of RFB-SVM-RFE is its peculiar insight on the individual feature contribution to classification accuracy, SP, and SE. The feature ranking reported in Figure 3 highlighted that the combination of features from different domains is effectively enhancing the model discriminative performance compared to traditional univariate analysis. Specifically, the top three features encompassed *Cd*, *Fd*, and *MTd* domains, respectively. This finding supports the notion that IUGR condition is effectively impairing the fetal ANS under different aspects; thus a comprehensive set of attributes are required for an accurate determination of such pathological condition. Moreover, the presented methodology allowed evaluating the contribution of each feature in terms of SE and SP as reported in Figure 4. If the SE contributes appeared moderately distributed among the features included in the model, this was not verified for SP. This figure of merit achieved the optimal performance only when all the selected attributes were included in the SVM framework. It is possible to speculate that the described behavior is a consequence of the grid search design. Specifically, the requirement of SE maximization allowed achieving an adequate SP at the expense of its robustness.

An additional advantage of RFB-SVM is the opportunity to define the relationship between features as nonlinear; thus, it allows overcoming the limitation of linearity imposed by traditional SVM approaches. At the same time, despite increasing the overall complexity of classification if compared to more traditional SVM implementations, the radial kernel tuning is on average of reduced complexity with respect to polynomial kernel given the fewer hyperparameters to be optimized. Lastly, RBF kernel is mathematically more stable in contrast to polynomial kernel which tends to converge to either infinity or zero for larger degrees (Hsu et al., 2003).

To our knowledge, this work is the first attempt toward a CTG and quantitative feature-based discrimination of late IUGR condition. Previous research mainly focused on the investigation of animal models (Poudel et al., 2015) and analyses of metabolic (Sanz-Cortés et al., 2013) and Doppler profiles (Parra-Saavedra et al., 2013) of chronic hypoxia in the fetal period. Nevertheless, the underpinning and widely reported consequence of long-lasting oxygen deprivation is responsible for a delay in the maturation of the branches of ANS and their subsequent integration with the central nervous system (CNS). The impairment in ANS maturation was consistently found in this investigation by various quantitative CTG-derived parameters which have been extensively associated with the fetal ANS modulation throughout pregnancy as standalone features (Signorini et al., 2003; Gonçalves et al., 2018). In comparison with previous machine learning-derived and univariate results by our group in different populations of early IUGR (Ferrario et al., 2007; Fanelli et al., 2013; Signorini et al., 2020b), it is possible to observe a consistent discriminative power of features *LZC*, *HF_pow*, and *LTI*. Specifically, the average value of each feature was greater in the control group vs. late IUGR fetuses. On the other hand, we also report an enhanced classification contribution of *SampEn*, which outperformed *ApEn*. Lastly, in the described late IUGR population, short scale (*T* = *s* = 5 and *T* = *s* = 9) PRSA-extracted features were characterized by a greater discriminative power compared to global ones (*T* = 40 and *s* = 1). The reported findings are in accordance with the univariate results and support the hypothesis of an impaired fetal beat-to-beat responsiveness regulation in the context of nutrient restriction and chronic hypoxemia (Fanelli et al., 2013; Rivolta et al., 2019). Lastly, toward enhancing the general applicability as well as interpretability of the proposed RBF-SVM model, we tested its performance once excluding the information of fetal sex. The knowledge about the sex of the fetus is banned in several countries across the globe; thus a fetal sex-independent model is expected to achieve wider applicability. Additionally, the influence of sex of the fetus over several of the physiology based heart rate features is object of open debate in the scientific community. Results showed nonstatistically different performances of fetal sex-removed RBF-SVM compared to the reference. Specifically, classification accuracy, sensitivity, and specificity were equal to 0.9208 (0.9012, 0.9413), 0.9247 (0.9018, 0.9493), and 0.7905 (0.7492, 0.8322); 0.8077 (0.7187, 0.8784), 0.8125, and 0.8000 in the training/testing and validation sets, respectively.

## Conclusion

This contribution aims at promoting the application of machine learning methodologies in the context of fetal and perinatal medicine, following the growing trend of the artificial intelligence application in medicine (Topol, 2019; Ghassemi et al., 2019). The presented approach demonstrated the reliability of an SVM inspired framework, encompassing the automatic selection of a subset of CTG-derived features, a satisfactory classification performance in terms of AUC, SE, and SP in both the training and validation sets, and interpretable set results suitable to be translatable in the clinical environment. Findings reported in this investigation support the importance of multivariate approaches to investigate the variety of implications resulting from a pathological condition such as late IUGR.

Despite satisfactory and promising classification performance, improvements may be envisioned under various aspects. First, the inclusion of additional features such as the ones inspired to multiscale and fractal analysis might further contribute to classification accuracy as reported in the context of intrapartum (Spilka et al., 2017). Second, the performance of different machine learning approaches as well as deep learning methodologies should be investigated and compared to RFB-SVM-RFE. Lastly, the validation of the presented approach should be carried out on external datasets to ultimately test the model performance as a function of different reference values of the input features. Additionally, it would be relevant to evaluate the validity of the proposed model in the context of the early insurgence of the pathology. A recent dataset of FHR indices extracted from a population of early IUGR fetuses can be found in Signorini et al. (2020a).

## Data Availability

Publicly available datasets were analyzed in this study. This data can be found here: IEEE DataPort—https://ieee-dataport.org/open-access/fetal-heart-rate-features-healthy-and-late-iugr-fetuses.
